# A role for caspase‐2 in sphingosine kinase 1 proteolysis in response to doxorubicin in breast cancer cells – implications for the CHK1‐suppressed pathway

**DOI:** 10.1002/2211-5463.12344

**Published:** 2017-12-08

**Authors:** Brittany L. Carroll, Joseph Bonica, Achraf A. Shamseddine, Yusuf A. Hannun, Lina M. Obeid

**Affiliations:** ^1^ Department of Medicine Stony Brook Cancer Center Health Sciences Center Stony Brook University NY USA; ^2^ Northport Veterans Affairs Medical Center NY USA

**Keywords:** breast cancer, caspase, DNA damage response, p53, sphingolipid, sphingosine kinase

## Abstract

Sphingosine kinase 1 (SK1) is a lipid kinase whose activity produces sphingosine 1‐phosphate, a prosurvival lipid associated with proliferation, angiogenesis, and invasion. SK1 overexpression has been observed in numerous cancers. Recent studies have demonstrated that SK1 proteolysis occurs downstream of the tumor suppressor p53 in response to several DNA‐damaging agents. Moreover, loss of SK1 in p53‐knockout mice resulted in complete protection from thymic lymphoma, providing evidence that regulation of SK1 constitutes a major tumor suppressor function of p53. Given this profound phenotype, this study aimed to investigate the mechanism by which wild‐type p53 regulates proteolysis of SK1 in response to the DNA‐damaging agent doxorubicin in breast cancer cells. We find that p53‐mediated activation of caspase‐2 was required for SK1 proteolysis and that caspase‐2 activity significantly alters the levels of endogenous sphingolipids. As p53 is mutated in 50% of all cancers, we extended our studies to investigate whether SK1 is deregulated in the context of triple‐negative breast cancer cells (TNBC) harboring a mutation in p53. Indeed, caspase‐2 was not activated in these cells and SK1 was not degraded. Moreover, caspase‐2 activation was recently shown to be downstream of the CHK1‐suppressed pathway in p53‐mutant cells, whereby inhibition of the cell cycle kinase CHK1 leads to caspase‐2 activation and apoptosis. Indeed, knockdown and inhibition of CHK1 led to the loss of SK1 in p53‐mutant TNBC cells, providing evidence that SK1 may be the first identified effector of the CHK1‐suppressed pathway.

**Abbreviations**
BIFC: bimolecular fluorescence complementation
MEFs: mouse embryonic fibroblasts
S1P: sphingosine 1‐phosphate
SK1: sphingosine kinase 1
TNBC: triple‐negative breast cancer cells

Bioactive sphingolipids are recognized as important signaling molecules that play significant roles in a diverse array of biological processes, from cell death and senescence to cell survival and proliferation 1, 2. Sphingolipid metabolism is a multifaceted and interconnected network of many enzymes that are responsible for coordinating the production of bioactive lipids that at times may elicit very opposing effects within the cell; therefore, the activity of the enzymes in this network must be tightly regulated.

Sphingosine kinase 1 (SK1) is an important sphingolipid enzyme that is involved in maintaining the balance between the prosurvival lipid product of its kinase activity, sphingosine 1‐phosphate (S1P), and the upstream proapoptotic sphingolipid metabolites, ceramide and sphingosine. Therefore, alterations in SK1 activity could have a critical impact on cell fate. In line with this, previous work from our laboratory showed that SK1 mRNA and protein levels are significantly increased in numerous types of human cancers 3, 4. Subsequent complementary work from several other laboratories has demonstrated that deregulation of the SK1/S1P pathway plays a role in carcinogenesis and promoting cancer cell viability as well as chemotherapeutic resistance 5, 6, 7, 8, 9. In addition, there is an emerging body of literature implicating SK1 as a critical downstream target of the tumor suppressor p53 in response to DNA damage. Previous work from our laboratory showed that actinomycin D (Act‐D) induces SK1 degradation in Molt‐4 T‐cell leukemia cells, and this degradation is rescued in Molt‐4 cells overexpressing the papilloma virus E6 protein, which targets p53 to degradation, suggesting that SK1 proteolysis is p53 mediated. This work also showed that pretreatment of Molt‐4 cells with the pan‐caspase inhibitor Z‐VAD rescued SK1 degradation in response to Act‐D, suggesting that SK1 proteolysis is caspase mediated 10. Building on this work, Heffernan‐Stroud *et al*. showed that UVC‐induced SK1 proteolysis in WT mouse embryonic fibroblast (MEFs) is abrogated in p53‐null MEFs (p53−/−) 11. This led to the hypothesis that SK1 could be deregulated (i.e., does not get proteolyzed) in p53‐null tissues. Indeed, in that study, it was also demonstrated that SK1 was overexpressed and more active in the thymus of p53‐null mice. Moreover, deletion of SK1 in p53‐null mice completely abrogated thymic lymphomas in the double‐knockout mice and prolonged lifespan by approximately 30% compared to p53−/− mice with SK1 11.

The above data provide strong evidence identifying SK1 as an important downstream target of the tumor‐promoting activity in p53‐null mice, although many questions still remain as to the exact mechanism of SK1 proteolysis in cancer cells in response to DNA damage, and whether this pathway is perturbed in p53‐mutant cells. As a corollary, much is unknown about the biological implications of SK1 loss in p53‐mutant cancer.

Although some evidence in the literature indicates that SK1 proteolysis in response to DNA damage may be caspase mediated 10, 11, these studies utilized caspase inhibitors that lack the specificity to monitor the activity of a specific caspase as the common peptide sequences used have been demonstrated to inhibit many members of the caspase family 12. Moreover, p53 activity is well documented to be involved in the activation of a number of caspases 13, 14; nevertheless, a strong connection between p53 and the poorly studied caspase‐2 is emerging via its activation platform, the PIDDosome 15. The PIDDosome consists of three proteins, PIDD [p53‐induced death domain (DD) protein] that interacts with RAIDD (RIP‐associated Ich‐1/CED homologous protein with DD) via their DDs and caspase‐2 which is recruited to RAIDD via the caspase recruitment domain (CARD) present in both proteins. Once assembled, full‐length caspase‐2 can then undergo autoproteolytic cleavage producing the fully active enzyme 15.

To date, only 16 substrates of caspase‐2 have been identified 16, whose physiological functions range from vesicular trafficking and translation initiation to cell cycle regulation and apoptosis. Importantly, many of these substrates are also substrates of other caspases. In addition, caspase‐2−/− mice are viable, fertile, and show minimal phenotype other than deficiencies in apoptosis in oocytes, and accelerated apoptosis in motor and sympathetic neurons 17, 18. Interestingly, a novel caspase‐2‐dependent apoptotic pathway termed the CHK1‐suppressed pathway was recently identified, where loss or inhibition of the cell cycle checkpoint kinase CHK1 in the presence of mutant p53 promotes a caspase‐2‐mediated apoptotic response to DNA damage. However, critical downstream targets and components of this pathway are yet to be identified 19, 20, 21.

In light of the above data, we hypothesized that caspase‐2 may mediate p53‐dependent SK1 proteolysis in response to DNA damage. To test this hypothesis, we evaluated whether SK1 proteolysis is downstream of caspase‐2 activation using a variety of methods in WT p53 human breast cancer cells and also whether caspase‐2 is required for SK1 proteolysis using both biochemical and genetic models. Given that p53 mutations occur in 50% of all human cancers 22, 23, 24 and in a high proportion of triple‐negative breast cancer (TNBC) cells 25, we next wanted to extend these studies to investigate whether caspase‐2 activation and SK1 proteolysis are deregulated in these situations and if so, whether SK1 proteolysis can be restored by activation of the CHK1‐suppressed pathway in p53‐mutant TNBC cells.

## Experimental procedures

### Chemicals and reagents

Lipofectamine^®^ RNAiMAX, Annexin‐V, and propidium iodide were purchased from Life Technologies (Grand Island, NY, USA). X‐tremeGENE 9 DNA Transfection Reagent was purchased from Roche Diagnostics (Indianapolis, IN, USA). iTAQ and SYBR^®^ Green master mix master mix was purchased from Bio‐Rad (Hercules, CA, USA). CHK1/2 inhibitor AZD7762 was purchased from sSelleckchem (Houston, TX, USA). Doxorubicin hydrochloride and cycloheximide were purchased from Sigma Aldrich (St. Louis, MO, USA). D‐*Erythro*‐sphingosine (C17 base) was purchased from Avanti Polar Lipids (Alabaster, AL, USA). Thiazolyl blue tetrazolium bromide (MTT) was purchased from Amresco (Solon, OH, USA). SKi‐II (4‐[4‐(4‐chloro‐phenyl)‐thiazol‐2‐ylamino]‐phenol) was purchased from Cayman Chemical (Ann Arbor, MI, USA).

### Cell culture and siRNA

MCF7 and MDA‐MB‐231 cells were purchased from ATCC and cultured in RPMI 1640 medium and Dulbecco's modified Eagle's medium (DMEM), respectively, with 10% FBS both from Life Technologies (Grand Island, NY). WT and Cas2−/− MEFs were a kind gift from D. Green (St. Jude's Children Hospital) and cultured in DMEM with 10% FBS both from Life Technologies (Grand Island, NY, USA). Before all doxorubicin treatments, the media were changed on cells to fresh new media. Gene silencing was then carried out using siRNA directed against human SK1 (target sequence 5′‐AAGGGCAAGGCCTTGCAGCTC‐3) and all‐star siRNA as a negative control purchased from Qiagen. The siRNA directed against CHK1 and p53 were validated predesigned sequences from Invitrogen. Transfections were carried out using Lipofectamine^®^ RNAiMAX from Life Technologies (according to the manufacturer's protocol). For siRNA experiments, cells were seeded into 60‐mm plates at < 75 000 cells per dish and treated with 20 nm siRNA for 48 h prior to stimulation.

### RNA isolation and quantitative RT‐PCR

RNA extraction and cDNA synthesis were carried out using PureLink^®^ RNA Mini Kit (Life Technologies) and Quanta qScript cDNA SuperMix (Quanat Biosciences, Beverly, MA, USA), respectively, and according to the manufacturer's instructions. The cDNA was then diluted (1 : 15) in RNase‐free water, and 5 μL was used in a total reaction volume of 20 μL. For each 20‐μL real‐time PCR, a ratio of 10 : 1 : 4 [iTaq: Taqman probe (20×): nuclease‐free water] was used. PCR was performed using the Applied Biosystems 7500 Real‐Time PCR System (Applied Biosystems, Foster City, CA, USA). The following Taqman probes (Life Technologies) were used: mouse SK1 (Mm00448841_g1) and mouse SK2 (Mm00445021_m1). Cycle threshold (*C*_t_) values were obtained for each gene of interest and *β*‐actin. Δ*C*_t_ values were calculated and the relative gene expression normalized to control samples was calculated from ΔΔ *C*_t_ values.

### Western blot analysis

Cultured or transfected cells were washed with ice‐cold PBS and then directly lysed in cold RIPA buffer containing 1 mm sodium orthovanadate, 2 mm PMSF, and protease inhibitor cocktail (Santa Cruz Biotechnology, Santa Cruz, CA, USA). Cellular lysates were then clarified by centrifugation at 18 800 ***g*** for 10 min at 4 °C; protein concentration was quantitated by BCA Protein Assay kit from Thermo Scientific (Suwanee, GA, USA). Equal amounts of protein (25 μg) were boiled in Laemmli buffer (Boston Bio Product) and separated on SDS/PAGE (4–15%, Tris/HCl) using the Bio‐Rad Criterion system. Separated proteins were then transferred onto nitrocellulose membranes (Bio‐Rad) and blocked with 5% nonfat milk in PBS‐0.1% Tween‐20 (PBS‐T) for 1 h at room temperature. Primary antibodies diluted 1 : 1000 or 1 : 20 000 for *β*‐actin and GADPH were then added to membranes and incubated at 4 °C overnight. Membranes were washed three times with PBS‐T, then incubated with diluted 1 : 5000 HRP‐conjugated secondary antibodies for 1 h at room temperature. Membranes were then washed for 1 h, incubated with Pierce ECL Western Blotting Substrate (Pierce, Waltham, MA, USA), and exposed to X‐ray films that were then processed and scanned. Anti‐SK1, anti‐total CHK1, anti‐phospho (296)‐CHK1, anti‐p53, anti‐caspase‐2, and anti‐GAPDH were from Cell Signaling Technology (Danvers, MA, USA). RIPA lysis buffer system, HRP‐labeled secondary antibodies, and anti‐PARP were from Santa Cruz Biotechnology. Anti‐CERT was from Bethyl Laboratories (Montgomery, TX, USA). Anti‐caspase‐2 (clone 11B4) was from Millipore (Billerica, MA, USA).

### Sphingolipidomic analysis

Following the indicated treatment, cells were directly lysed with 2 mL of 2 : 3 ratio of 70% isopropanol/ethyl acetate, followed by the gentle scraping of the cell from the culture plate. Lysate was then transferred to 15‐mL falcon tubes. Upon the addition of internal standards to the tubes, samples were then briefly centrifuged at 2000 ***g*** and the upper phase was transferred to a new glass tube. An additional round of extraction was performed on the remaining volume. After combining the two extracts, sphingolipids and inorganic phosphates were measured by the Lipidomics Core Facility at the Stony Brook University of New York using HPLC/MS determination of sphingolipid mass levels as described previously 26.

### C17‐Sph labeling

Cells were plated at ~ 150 000 cells/60‐mm dish. Fifteen minutes prior to the end of treatment time, cells were incubated with 1 μm C17 sphingosine for the remaining 15 min. The cells were then washed with PBS, and 2 mL of cell extraction mixture (2 : 3 70% isopropanol/ethyl acetate) was then directly added to the cells. The cells were then gently scraped and extracts were sent for analysis at the Lipidomics Core Facility of Stony Brook University Medical Center as described above and previously 26.

### Bimolecular fluorescence complementation

As described previously 27, 28, ~ 75 000 cells were grown on poly‐d‐lysine‐coated 35‐mm confocal dishes (MatTek Corporation, Ashland, MA, USA) overnight. The following day, cells were transiently transfected with C2‐CARD VN (500 ng) and C2‐CARD VC (500 ng) along with pshooter.dsRed‐mito (250 ng) as a reporter for transfection. Twenty‐four hours after transfection, cells were treated with doxorubicin for 24 h, and then, the percentage of pshooter.ds.Red‐mito‐positive (red) cells that were Venus positive (green) was determined from a minimum of 100 cells per plate. Live cell imaging was conducted using a Leica TCS SP8 scanning‐laser confocal microscope in a chamber at 37 °C and 5% CO_2_ (Leica, Wetzlar, Germany). The plasmids pBIFC‐C2‐CARD VC and pBIFC‐C2‐CARD VN were kindly provided by D. Green (St. Jude's Children Hospital). pDsRed‐Mito was purchased from Clontech (Mountain View, CA, USA).

### Flow cytometric analysis of apoptosis

Apoptotic cells were detected by Annexin‐V/propidium iodide (PI) staining using Alexa Fluor^®^ 488 Annexin V and PI detection kit (Life Technologies) according to the manufacturer's protocol. Briefly, after the indicated treatment, cells were trypsinized, collected by brief centrifugation, and washed with ice‐cold PBS. Cells were then resuspended in buffer containing Alexa Fluor^®^ 488 Annexin‐V and PI (at concentrations indicated in the manufacturer's protocol) for 15 min at room temperature and in the darkness. After incubation, cells were immediately analyzed using a Becton Dickinson FACSCalibur. Ten thousand events were acquired on the FACSCalibur (Becton Dickinson Biosciences, San Jose, CA, USA) and followed by analysis with cellquest (Becton Dickinson) software.

### MTT assay

Cells were plated at ~ 150 000 cells per well in a six‐well plate. After indicated treatment, media were replaced with 1 mL of fresh media with 1 mL of 12 mm MTT (Amresco) solution and incubated at 37 °C in the dark for 30 min. The media/MTT mixture was then replaced with 2 mL of DMSO and incubated with the gentle rocking for 10 min. Following incubation, 200 μL from each well in triplicate was then assayed in 96‐well plate using a spectrophotometer at 570 nm.

### Trypan blue cell death assay

Cells were plated at ~ 250 000 cells per plate on 6‐cm plates. After treatment with 0.8 μm doxorubicin, media were removed and put into a 15‐mL conical Falcon tube, and cells were trypsinized at specific time points. After spinning the cells down at 100 ***g*** for 5 min, the cells were resuspended in the old media, and 20 μL of a 1 : 1 dilution of media and trypan blue was prepared. The mixture was left at room temperature for 5 min, and the cells were counted on a hemacytometer. Cells with a blue center were considered dead, while those with a clear center were considered live. The percentage of live cells was calculated as a number of total cells.

### Caspase activity assay

Caspase‐3 activity was measured using BioVision (Milpitas, CA, USA) Caspase‐Family Fluorometric Substrate Set according to the manufacturer's protocol. Briefly, ~ 150 000 cells/well were plated in a six‐well plate. The following day, cells were treated accordingly in addition to control cells. Cells were lysed with 50 μL of cell lysis buffer, and protein concentration was determined by BCA Protein Assay kit from Thermo Scientific. In a 96‐well plate, a total of 50 μg of cell lysate in a volume of 50 μL was added to 50 μL of 2× reaction buffer containing 10 mm DTT and 5 μL of the AFC‐conjugated caspase‐3 substrate, AC‐DEVD‐AFC. Samples were incubated at 37 °C in the dark for 1 h and then read by a fluorometer equipped with a 400‐nm excitation filter and 505‐nm emission filter.

### Statistical analysis

The data are represented as the means ± SE Unpaired Student's *t*‐test and two‐way ANOVA with Bonferroni post‐test, statistical analyses were performed using prism/graphpad software (Prism, La Jolla, CA, USA).

## Results

### P53‐mediated SK1 proteolysis is downstream of caspase‐2 activation

Doxorubicin is an anthracycline frequently used in the treatment for breast cancer. It is a well‐known inducer of p53 29, has been shown to initiate p53‐dependent SK1 proteolysis 10 and to induce apoptosis in a number of cell lines via caspase activation 22, 29. Several studies have demonstrated caspase‐2 activation upon doxorubicin treatment in numerous cell systems including leukemia cells, MEFs, and mouse oocytes. These studies also showed that loss of caspase‐2 in these cells results in significantly reduced sensitivity to doxorubicin compared with control cells 17, 30, albeit effects in breast cancer cells have not been studied. We therefore first set out to determine whether doxorubicin activates caspase‐2 in wild‐type p53 MCF7 breast cancer cells and also whether SK1 proteolysis is downstream of caspase‐2 activation.

**Figure 1:**
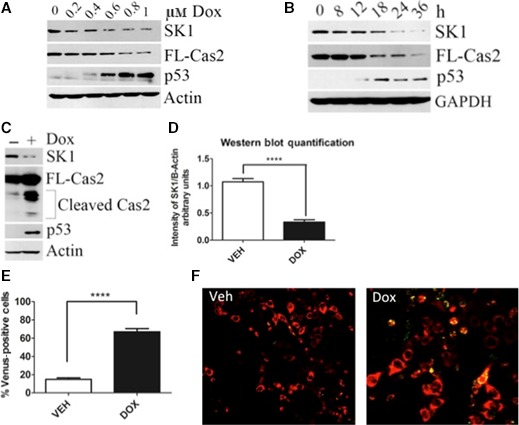
SK1 proteolysis is downstream of caspase‐2 upon doxorubicin treatment in MCF7 breast cancer cells. (A) MCF7 cells were treated with the indicated dose of doxorubicin for 24 h. Cells were then harvested in RIPA buffer, and total cell lysate was analyzed by western blot for the proteins indicated. (B) MCF7 cells were treated with 0.8 μm doxorubicin for the indicated times and harvested, and total cell lysate was analyzed by western blot for the proteins indicated. (C) MCF7 cells were treated with 0.8 μm doxorubicin for 24 h and harvested in RIPA buffer, and total cell lysate was analyzed by western blot for the proteins indicated. (D) imagej was used to quantify SK1 protein levels normalized to actin from (C) and all replicates (*n* = 6 ****P < 0.005 by Student's *t*‐test). (E) MCF7 cells were transiently transfected with C2‐CARD VN (500 ng) and C2‐CARD VC (500 ng) along with pshooter.dsRed‐mito (250 ng) as a reporter for transfection. Twenty‐four hours after transfection, cells were treated with 0.8 μm doxorubicin for 24 h and then the percentage of pshooter.ds.Red‐mito‐positive (red) cells that were Venus positive (green) was determined from a minimum of 100 cells per plate. Data are presented as mean ± SEM of three independent experiments (*n* = 3 ****P < 0.001 by Student's *t*‐test). Representative confocal images of cells from (F) are shown.

A dose response of doxorubicin revealed that with increasing dose from 0.2 μm to 1 μm, p53 accumulation was followed by a significant reduction in procaspase‐2 that was concomitant with the loss of SK1 at the protein level (Fig. 1A). These data are consistent with previous studies showing that loss of SK1 by genotoxic stress is a post‐translational event 10, 11. Similarly, a time course using 0.8 μm doxorubicin showed a substantial accumulation of p53 at 18 h, corresponding with a significant processing of full‐length caspase‐2 followed by almost complete loss of SK1 protein by 24 h (Fig. 1B). To further validate the activation of caspase‐2, 0.8 μm doxorubicin was used at 24 h to monitor the cleavage of full‐length caspase‐2 into its active fragments by western blot as well as dimerization of caspase‐2 as measured by bimolecular fluorescence complementation (BIFC) 28. As shown in Fig. 1C, there was a significant accumulation of the cleaved form of caspase‐2 concurrent with an approximate 80% reduction in SK1 at the protein level, as quantified in Fig. 1D. In accordance with the western blot data, caspase‐2 activation was observed in approximately 60% of transfected cells counted at 24 h after doxorubicin treatment as measured by BIFC (Fig. 1E). Representative images from the BIFC experiment are shown in Fig. 1F. Taken together, these results show the activation of caspase‐2 in response to doxorubicin in a time frame corresponding to the loss of SK1. To determine what effect caspase‐2 activation had on cell death, a trypan blue assay was conducted on cells treated in a time course with 0.8 μm of doxorubicin. This assay showed no difference in cell death between vehicle‐ and doxorubicin‐treated cells until 36 h post‐treatment (Fig. S6).

### Doxorubicin significantly alters sphingolipid metabolism

**Figure 2:**
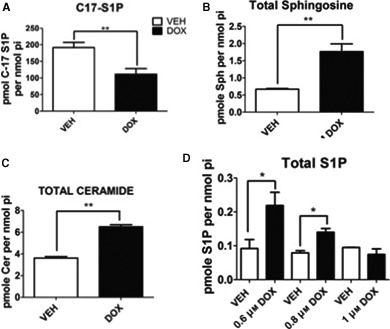
Effects of doxorubicin on SK activity and endogenous sphingolipids. (A) MCF7 cells were treated with 0.8 μm doxorubicin for 24 h and then incubated with C_17_‐sphingosine for 15 min. Following incubation, cells were harvested for sphingolipidomic analysis by liquid chromatography/mass spectrometry and the C_17_‐containing S1P was normalized to the amount of lipid phosphate for each sample (*n* = 3 **P < 0.01 by Student's *t*‐test). MCF7 cells were treated with 0.8 μm doxorubicin for 24 h and then harvested for sphingolipidomic analysis as in (A) to measure sphingosine (B) and total ceramides (C) (*n* = 3 **P < 0.01 by Student's *t*‐test). (D) MCF7 cells were treated with the indicated dose of doxorubicin for 24 h, harvested as in (A) to measure S1P (*n* = 3 *P < 0.05 by Student's *t*‐test).

Next, to investigate whether doxorubicin‐induced SK1 proteolysis results in a decrease in SK activity, the incorporation of C_17_‐sphingosine into C_17_‐S1P was measured. As shown in Fig. 2A, upon doxorubicin treatment, there was approximately a 50% reduction in the incorporation of C_17_‐sphingosine into C_17_‐S1P, demonstrating attenuation of ongoing SK activity. In agreement with this reduction in SK activity, a significant increase in SK's endogenous substrate sphingosine was observed (Fig. 2B), as well as a 1.5‐fold increase in total ceramide (Fig. 2C). Although SK activity was significantly decreased (Fig. 2A), we observed an increase in endogenous S1P at 0.8 μm upon doxorubicin treatment. To further investigate S1P levels upon doxorubicin treatment, we performed a dose response from 0.6 to 1 μm. Interestingly although S1P levels are increased at 0.8 μm doxorubicin, they are decreased from 0.6 μm doxorubicin, a dose where SK1 is not proteolyzed and S1P levels decreased further at a higher dose of 1 μm doxorubicin (Fig. 2D). S1P levels are maintained in the cell via the action of SK1 and SK2, which are responsible for the production of S1P, and S1P lyase and phosphatases, which are responsible for its breakdown. Therefore, these results can be explained through the action of SK2 or delayed breakdown of S1P by the lyase or phosphatase. These results demonstrate a biochemical and functional role for the effects of doxorubicin on SK1 activity and the levels of key bioactive sphingolipids.

### Caspase‐2 activation and SK1 proteolysis are p53 mediated

**Figure 3:**
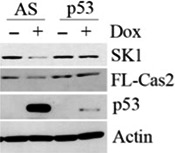
P53‐mediated SK1 proteolysis and activation of caspase‐2. MCF7 cells were transfected with p53 siRNA (20 nm). Forty‐eight hours after transfection, cells were treated with 0.8 μm doxorubicin for 24 h and harvested and total cell lysate was analyzed by western blot for the proteins indicated.

To confirm that caspase‐2 activation and subsequent SK1 proteolysis are p53 mediated, we depleted p53 in MCF7 cells by siRNA. Indeed, loss of p53 abrogated both processing of full‐length caspase‐2 and proteolysis of SK1 (Fig. 3).

These data indicate for the first time and using a variety of different methods that the chemotherapeutic doxorubicin was an inducer of caspase‐2 activation in MCF7 breast cancer cells. These data also provide strong evidence that SK1 proteolysis is accompanied by caspase‐2 activation in response to doxorubicin in a time‐ and dose‐dependent manner, both of which are abrogated in the absence of p53.

### Caspase‐2 is required for SK1 proteolysis in response to DNA damage

**Figure 4:**
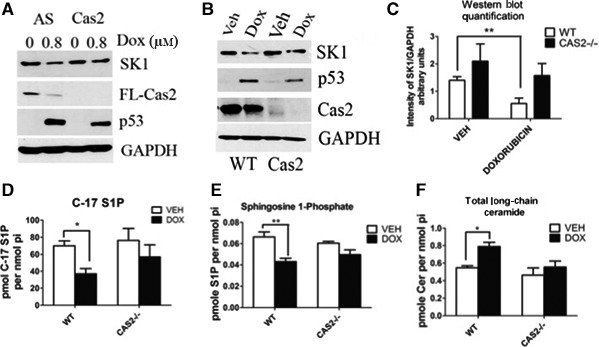
Caspase‐2 is required for SK1 proteolysis in response to DNA damage. (A) MCF7 cells were transfected with caspase‐2 siRNA (20 nm) or control siRNA (AS). Forty‐eight hours after transfection, cells were treated with 0.8 μm doxorubicin or vehicle for 24 h and harvested, and total cell lysate was then analyzed by western blot for the proteins indicated. (B) MEFs expressing wild‐type caspase‐2 or with caspase‐2 knocked out were treated with 0.2 μm doxorubicin for 24 h and harvested, and total cell lysate was analyzed by western blot for the proteins indicated. (C) imagej was used to quantify SK1 protein levels normalized to GAPDH from (B) and all replicates (*n* = 4 **P < 0.01 by two‐way ANOVA). (D) MEFS were treated with 0.2 μm doxorubicin for 24 h and then incubated with C_17_‐labeled sphingosine for 15 min. Following incubation, MEFs were harvested for sphingolipidomic analysis by liquid chromatography/mass spectrometry, and the C_17_‐containing S1P was normalized to the amount of lipid phosphate for each sample (*n* = 3 *P < 0.05 by two‐way ANOVA). MEFs were treated with 0.2 μm doxorubicin and then harvested for sphingolipidomic analysis as in (D) to measure S1P (*n* = 3 **P < 0.01 by two‐way ANOVA) (E) and ceramide (*n* = 3 *P < 0.05 by two‐way ANOVA) (F).

Next in order to build on the findings of a mechanistic connection between genotoxic stress, p53, caspase‐2 activation, and SK1 proteolysis, we set out to investigate whether caspase‐2 is required for SK1 degradation. To this end, we employed two methods, a siRNA approach in MCF7 cells and a genetic model using MEFs expressing wild‐type caspase‐2 (WT) or with caspase‐2 knocked out (Cas2−/−). As shown in Fig. 4A, depletion of caspase‐2 by siRNA significantly abrogated doxorubicin‐induced SK1 proteolysis compared with All‐Star control siRNA‐transfected cells. Next, to ensure there was an effect on SK1 proteolysis, we observed that caspase‐2 knockdown was not due to off‐target effects of caspase‐2 siRNA and we investigated SK1 proteolysis in WT and Cas2−/− MEFs. In accordance with the caspase‐2 siRNA data, doxorubicin treatment resulted in a significant reduction in SK1 protein that was reversed by genetic deletion of caspase‐2 (Fig. 4B,C). Analysis of SK1 mRNA levels showed no significant changes in WT or Cas2−/− MEFs after doxorubicin (Fig. S1A), while there was a slight reduction in SK2 mRNA levels in both the WT and Cas2−/− MEFs (Fig. S1B), providing further evidence that the reduction in SK1 protein is a post‐translational event. To further investigate the regulation of SK1 in WT and Cas2−/− MEFs, we analyzed protein stability of SK1 in the MEFs using the protein synthesis inhibitor cycloheximide. Interestingly, in the WT MEFs within 12 h after treatment with cycloheximide, SK1 protein was reduced by approximately 30% compared with vehicle‐treated MEFs, whereas there was no significant change in SK1 levels in the Cas2−/− MEFs (Fig. S1C). Only after 30 h of cycloheximide treatment, there was a reduction in SK1 protein level of approximately 40% observed in the Cas2−/− MEFs compared with vehicle‐treated MEFs (Fig. S1C), indicating that SK1 protein is likely somewhat more stable in the Cas2−/− MEFs compared with WT MEFs.

Accordingly, we sought to investigate the effects loss of caspase‐2 has on sphingolipid metabolism. To accomplish this, we first measured SK activity in WT and Cas2−/− MEFs. Consistent with the observations at the protein level, the significant reduction in SK activity observed in the WT MEFs after doxorubicin treatment, as measured by the reduction in the incorporation of C17‐sphingosine into C17‐S1P, was not observed in the cas2−/− MEFs (Fig. 4D). A similar reduction in SK activity in WT MEFs that was abrogated in cas2−/− MEFs was also observed after UV irradiation (Fig. S1D). Of note, the levels of S1P were significantly reduced after doxorubicin treatment in WT MEFs (Fig. 4E) concomitant with a significant increase in proapoptotic total long‐chain ceramide species 31 (Fig. 4F). Both the decrease in S1P and increase in long‐chain ceramide species were abrogated in the Cas2−/− MEFs (Fig. 4E,F).

Collectively, these data provide strong evidence that caspase‐2 is required for SK1 proteolysis and that loss of caspase‐2 significantly affects endogenous sphingolipid levels in response to genotoxic stress. Importantly, these data support a novel and important role of caspase‐2 in regulating sphingolipid metabolism in response to cellular stress.

### SK1 is deregulated in p53‐mutant TNBC cells

**Figure 5:**
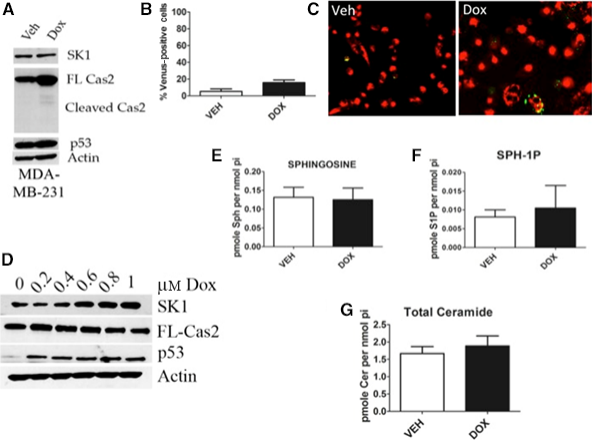
SK1 is deregulated in p53‐mutant TNBC cells in response to doxorubicin. (A) MDA‐MB‐231 cells were treated with 0.8 μm doxorubicin for 24 h and harvested in RIPA buffer, and total cell lysate was analyzed by western blot for the proteins indicated. (B) MDA‐MB‐231 cells were transiently transfected with C2‐CARD VN (500 ng) and C2‐CARD VC (500 ng) along with pshooter.dsRed‐mito (250 ng) as a reporter for transfection. Twenty‐four hours after transfection, cells were treated with 0.8 μm doxorubicin for 24 h, and then, the percentage of pshooter.ds.Red‐mito‐positive (red) cells that were Venus positive (green) was determined from a minimum of 100 cells per plate. Data are presented as mean ± SEM of three independent experiments. Representative confocal images of cells from (C) are shown. (D) MDA‐MB‐231 cells were treated with the indicated dose of doxorubicin for 24 h. Cells were then harvested in RIPA buffer, and total cell lysate was analyzed by western blot for the proteins indicated. (E) MDA‐MB‐231 cells were treated with 0.8 μm doxorubicin for 24 h and harvested for sphingolipidomic analysis by liquid chromatography/mass spectrometry, and the sphingosine (E), S1P (F), and ceramide (G) were normalized to the amount of lipid phosphate for each sample (*n* = 3).

Next, in order to investigate whether p53 mutations affect SK1 proteolysis and endogenous sphingolipid levels in response to genotoxic stress, we utilized the TNBC cell line MDA‐MB‐231 that harbors a missense mutation, R280K, in the DNA‐binding motif of p53. Interestingly, in contrast to MCF7 cells, doxorubicin treatment did not result in caspase‐2 activation as measured by cleavage of the proform of caspase‐2 (Fig. 5A,D) and by BIFC, with <20% of the total number of transfected cells counted positive for activated caspase‐2 (Fig. 5B,C). In accordance with caspase‐2‐mediated proteolysis of SK1 as described in Fig. 4, no significant reduction in SK1 protein level was observed in response to doxorubicin at any dose tested in MDA‐MB‐231 cells (Fig. 5A,D); conversely, an increase in SK1 protein level was observed with increasing doses of doxorubicin (Fig. 5D). Interestingly, the ceramide transport protein CERT was recently characterized as a caspase‐2 substrate in response to tumor necrosis factor‐α 32. Given that CERT is involved in sphingolipid homeostasis, we hypothesized that it would also be deregulated in this system. Indeed, in MCF7 cells when caspase‐2 is activated, cleavage of CERT was observed; caspase‐2 processing and cleavage of CERT were not detectable in UV‐irradiated MDA‐MB‐231 (Fig. S2A).

We next wanted to investigate the levels of endogenous sphingolipids after genotoxic stress in MDA‐MB‐231 cells. There was no significant change in the substrate or product of SK activity, sphingosine, and S1P, respectively (Fig. 5E,F). Furthermore, there was no significant increase in proapoptotic ceramide after doxorubicin treatment (Fig. 5G). Taken together, these results demonstrate that caspase‐2 activation is not achieved in p53‐mutant cells, and likewise, SK1 is not reduced, further cementing the relationship between p53, caspase‐2, and SK1. We also extend these findings to a previously identified caspase‐2 substrate and sphingolipid protein CERT.

### Loss of SK1 sensitizes p53‐mutant TNBC cells to doxorubicin

**Figure 6:**
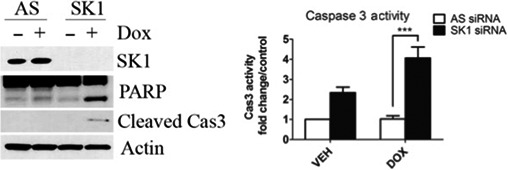
Effects of loss of SK1 on p53‐mutant TNBC cells in response to doxorubicin. (A) MDA‐MB‐231 cells were transfected with SK1 siRNA (20 nm). Sixty hours after transfection, cells were treated with 0.8 μm doxorubicin for 24 h and then harvested, and total cell lysate was analyzed by western blot for the proteins indicated. (E) MDA‐MB‐231 cells were transfected with SK1 siRNA (20 nm). Sixty hours after transfection, cells were treated with 0.8 μm doxorubicin for 24 h and harvested, and caspase 3 activity was measured (*n* = 3 ***P < 0.005 by two‐way ANOVA).

We next sought to determine the functional consequence of deregulation of SK1. We hypothesized that SK1 deregulation in p53‐mutant TNBC cells may contribute to enhanced survival in response to doxorubicin. To test this hypothesis, SK1 was depleted by siRNA to evaluate whether the loss of SK1 in combination with doxorubicin could sensitize MDA‐MB‐231 cells to DNA damage. Indeed, knockdown of SK1 in combination with doxorubicin resulted in significant PARP and caspase 3 cleavage (Fig. 6A) and an approximately twofold increase in caspase 3 activity (Fig. 6B) compared with doxorubicin alone. On the other hand, knockdown of SK1 in MCF7 breast cancer cells had no effect on the doxorubicin response (Fig. S3A). This is expected as SK1 protein levels are already significantly reduced in response to genotoxic stress in MCF7 cells and therefore knockdown provided no further sensitization.

Altogether, these data identify deregulation of SK1 proteolysis in the context of p53‐mutant TNBC cells as a possible mechanism of resistance to DNA damage in response to doxorubicin, and consequently, the loss of SK1 sensitized these cells to doxorubicin.

### SK1 is downstream of the CHK1‐suppressed pathway in p53‐mutant TNBC cells in response to doxorubicin

**Figure 7:**
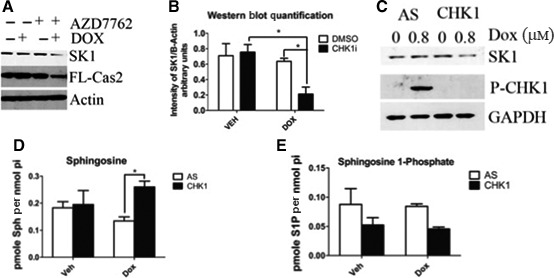
SK1 is downstream of the CS pathway in p53‐mutant TNBC cells in response to doxorubicin. (A) MDA‐MB‐231 cells were pretreated with 0.3 μm CHK1 inhibitor (AZD7762) for 2 h and then treated with 0.8 μm doxorubicin for 24 h and harvested, and total cell lysate was analyzed by western blot for the proteins indicated. (B) imagej was used to quantify SK1 protein levels normalized to actin from (A) and all replicates (*n* = 3 *P < 0.05 by two‐way ANOVA). (C) MDA‐MB‐231 cells were transfected with CHK1 siRNA (20 nm). Forty‐eight hours after transfection, cells were treated with 0.8 μm doxorubicin for 24 h and harvested and total cell lysate was analyzed by western blot for the proteins indicated. MDA‐MB‐231 cells were transfected with CHK1 siRNA (20 nm). Forty‐eight hours after transfection, cells were treated with 0.8 μm doxorubicin for 24 h and then harvested for sphingolipidomic analysis by liquid chromatography/mass spectrometry to measure sphingosine (D) or S1P (E) (*n* = 3 *P < 0.05 by two‐way ANOVA).

As mentioned previously, there is an emerging apoptotic pathway, the CHK1‐suppressed pathway that identifies loss or inhibition of CHK1 as being essential for caspase‐2 activation in p53‐mutant cells 19, 20; therefore, we reasoned that activation of the CHK1‐suppressed pathway in MDA‐MB‐231 cells would result in caspase‐2 activation and subsequent SK1 proteolysis in response to doxorubicin. Indeed, inhibition of CHK1, with the CHK1/2 inhibitor AZD7762, led to a reduction in full‐length caspase‐2 concomitant with a significant loss of SK1 at the protein level (Fig. 7A,B). As AZD7762 is also known to inhibit other kinases in addition to CHK1 including CHK2, we next depleted CHK1 by siRNA to evaluate the specific role of CHK1. As shown in Fig. 7C, knockdown of CHK1 in combination with doxorubicin resulted in a decrease in SK1 protein, in accordance with the data obtained with CHK1 inhibition.

We then endeavored to see whether SK1 protein levels could be rescued by inhibiting caspase‐2 action after activation of the CHK1‐suppressed pathway. To do so, we treated MDA cells with the caspase‐2 inhibitor Z‐VDVAD‐FMK after treatment with AZD7762. Z‐VDVAD‐FMK is an exclusive inhibitor of caspase‐2, and as such, any restoration of protein levels could be considered the result of caspase‐2 inhibition. MDA cells were pretreated with AZD7762 and Z‐VDVAD‐FMK prior to doxorubicin treatment. Indeed, protein levels of SK1 do appear to recover upon treatment with caspase‐2 inhibitor (Fig. S5A). While the difference is not statistically significant, there does appear to be a trend toward higher protein levels when caspase‐2 is inhibited (Fig. S5B).

Next, the functional effects of CHK1 loss and activation of the CHK1‐suppressed pathway on endogenous sphingolipid levels were evaluated in combination with doxorubicin treatment. As demonstrated in Fig. 7D, there was a significant increase in SK1's substrate sphingosine in response to the loss of CHK1 and doxorubicin compared with doxorubicin alone. Moreover, there was a decrease in SK1's product and prosurvival sphingolipid, S1P, upon CHK1 loss (Fig. 7E); although this decrease was not statistically significant, even small changes in this potent bioactive lipid can significantly affect cell fate. Also of note, under the same conditions, no significant increase in the upstream metabolite ceramide was detected (Fig. S4A). Taken together, these results demonstrate a role of CHK1 upstream of caspase 2 leading to the loss of SK1 and significant changes in bioactive sphingolipids.

### SK1 as an effector of the CHK1‐suppressed pathway of apoptosis

**Figure 8:**
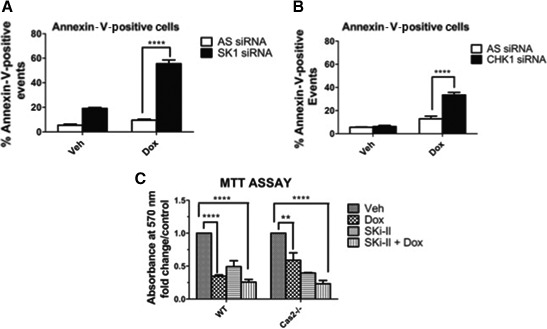
Loss of SK1 sensitizes p53‐mutant TNBC cells to a greater extent than loss of CHK1. (A) MDA‐MB‐231 cells were transfected with SK1 siRNA (20 nm). Sixty hours after transfection, cells were treated with 0.8 μm doxorubicin for 24 h, labeled with Annexin‐V, and analyzed by flow cytometry to detect apoptotic cells as described in ‘Experimental procedures’ (*n* = 3 ****P < 0.001 by two‐way ANOVA). (B) MDA‐MB‐231 cells were transfected with CHK1 siRNA (20 nm). Forty‐eight hours after transfection, cells were treated with 0.8 μm doxorubicin for 24 h, labeled with Annexin‐V, and analyzed by flow cytometry to detect apoptotic cells as described in ‘Experimental procedures’ (*n* = 3 ****P < 0.001 by two‐way ANOVA). (C) MEFs were pretreated with 10 μm Ski‐II for 1 h followed by 0.2 μm doxorubicin for 24 h. MTT assay was then performed as described in ‘Experimental procedures’ to assess cell viability.

Currently, there are numerous CHK1 inhibitors in a clinical trial as a combination therapy to treat cancers such as breast and ovarian 33, 34. Intriguingly, several studies and reports have indicated CHK1 inhibition as being very effective as a combination therapy to treat TNBC cells 35, 36. As we identified SK1 as being a target of the CHK1‐suppressed pathway, we wanted to investigate whether the loss of SK1 in MDA‐MB‐231 TNBC cells could have similar effects in promoting apoptosis. Remarkably, doxorubicin combined with the loss of SK1 resulted in roughly 60% of cells undergoing apoptosis as measured by Annexin‐V staining (Fig. 8A). This compares with only approximately 30% apoptotic cells when CHK1 was depleted (Fig. 8B).

In light of these results indicating that SK1 may be an effector of the caspase‐2 apoptotic response, we next wanted to determine whether inhibition of SK1 with the nonlipid SK inhibitor Ski‐II could also sensitize Cas2−/− MEFs to doxorubicin. Indeed, WT MEFs were very sensitive to doxorubicin, and as expected, inhibition of SK had no additive effects when combined with doxorubicin. In contrast, Cas2−/− MEFs displayed less sensitivity to doxorubicin than WT MEFs but when combined with SK1 inhibition, the Cas2−/− MEFs become as sensitive as WT (Fig. 8C).

Altogether, these data indicate SK1 as a novel downstream target of the CHK1‐suppressed pathway and provide evidence that loss of SK1 may be a crucial step in the CHK1‐suppressed pathway and caspase‐2‐mediated apoptosis. The data also provide evidence that loss of SK1 can specifically sensitize p53‐mutant TNBCs to a greater extent than CHK1 loss in addition to cells deficient in caspase‐2 to the cytotoxic effects of doxorubicin.

## Discussion

The aim of this study was to elucidate the mechanism of p53‐mediated SK1 proteolysis. Consolidating previous studies using nonspecific caspase inhibitors that hint at a role of this family of proteases in SK1 proteolysis, we demonstrate that caspase‐2 is required for SK1 degradation in human breast cancer cells and that loss of caspase‐2 significantly affects sphingolipid metabolism in response to DNA damage. Interestingly, in unraveling the mechanism of SK1 proteolysis in WT p53 cells, we then could identify perturbations of this pathway in mutant TNBC cells, where persistent SK1 levels and deregulation of sphingolipid metabolites in response to DNA damage were observed. We found a defect in caspase‐2 activation in p53‐mutant cells that could be overcome by CHK1 inhibition and activation of the CHK1‐suppressed pathway leading to SK1 proteolysis.

As much research is currently focused on unraveling the role caspase‐2 plays within the cell, this study adds to an emerging literature implicating caspase‐2 activity with the regulation of both lipid and sphingolipid metabolism 32, 37, 38. Our data indicate that caspase‐2 activity is required for SK1 proteolysis. This is important as SK1 holds a crucial position in sphingolipid metabolism, acting to maintain homeostasis between proapoptotic sphingolipids such as ceramide and the prosurvival sphingolipid S1P; therefore, deregulation of SK1 could greatly affect cell survival. In line with this, we show that inhibition of SK1 can sensitize Cas2−/− MEFs to similar levels as WT MEFs to doxorubicin. Interestingly, a recent study revealed that the microRNA mir‐708 directly downregulates caspase‐2 and this downregulation is necessary to induce carcinogenicity of bladder cancer 39. Moreover, SK1 levels are documented as increased in bladder cancer, and elevated SK1 levels are associated with poor prognosis in bladder cancer 40. As more studies begin to elucidate the role of caspase‐2 in cancer, it will be interesting to investigate how levels of sphingolipid proteins that are regulated by caspase‐2 such as SK1 and CERT and subsequent sphingolipid levels are altered in these cancers and whether these alterations play an important role in tumorigenesis or response to therapy. It is important to note that this study does not implicate caspase‐2 as the cleaving agent of SK1. Rather, we show that caspase‐2 activation is required for the proteolysis of SK1 demonstrated in the above‐mentioned data. Our laboratory has tried to determine whether caspase‐2 cleaves SK1 directly, but thus far there have been no results indicating this to be the case. Therefore, it is possible that caspase‐2 is upstream of SK1 proteolysis and is involved in activating other necessary proteins. The identity of those other proteins is currently unknown.

Although the current results indicate that caspase‐2 is required for SK1 proteolysis after DNA damage, we could not find evidence that SK1 is a direct substrate of caspase‐2. One plausible explanation for this comes from research describing the caspase‐2‐PIDDosome as an important factor in maintaining p53 levels and regulating p53 dynamics after DNA damage 41. Oliver *et al*. 41 cleverly demonstrated that DNA damage and PIDD‐induced activation of caspase‐2 result in Mdm2 cleavage, bolstering p53 stability and activity in a positive feedback loop, and alternatively, loss of caspase‐2 results in decreased p53 levels. We know from our research 10, 11 that SK1 proteolysis is dependent on p53; therefore, one could imagine that a threshold of p53 needs to be achieved in order to initiate SK1 proteolysis. Our data in fact show decreased levels of p53 with both caspase‐2 knockdown and in the cas2−/− MEFs compared with control cells, providing evidence that this decreased level of p53 could hypothetically be insufficient to drive SK1 proteolysis.

Finally, in this report, we identify SK1 as a novel target of the CHK1‐suppressed pathway. To our knowledge, this is the first potential effector of the CHK1‐suppressed pathway to be identified. CHK1 plays significant roles in maintaining cellular homeostasis and is indispensable for normal development as CHK1‐knockout mouse are embryonic lethal; therefore, repeated or high dose use of CHK1 inhibitors could have undesired side effects in patients 42, 43. As the CHK1‐suppressed pathway is an emerging apoptotic pathway whose activation by CHK1 inhibitors can sensitize p53‐mutant cells, it is of great clinical significance to identify targets that are effectors of this pathway that could also be potential targets for therapeutic intervention 19, 20, 21. In this study, we provide evidence that targeting SK1 offers an exciting potential therapeutic avenue for patients with altered p53 status, which has proven a challenging obstacle in cancer therapy for years.

## Author contributions

BLC, JB, and AAS designed and executed experiments. YAH and LMO directed the work and discussed progress. All the authors contributed to writing and editing.

## Supporting information


**Fig. S1**. Caspase 2 is required for SK1 proteolysis.
**Fig. S2.** CERT cleavage by caspase 2 is deregulated in mutant p53 TNBC.
**Fig. S3**. Loss of SK1 has no effect on cell viability in MCF7 breast cancer cells in combination with doxorubicin.
**Fig. S4.** Ceramide levels are not significantly affected by loss of CHK1.
**Fig. S5.** Inhibition of caspase 2 modestly restores SK1 protein levels upon activation of the CHK1‐suppressed cell death pathway.
**Fig. S6**. MCF‐7 cells affects cell death rates after 36 h of treatment.

## References

[1] Hannun YA and Obeid LM (2008) Principles of bioactive lipid signalling: lessons from sphingolipids. Nat Rev Mol Cell Biol 9, 139–150. 18216770 10.1038/nrm2329

[2] Pyne NJ and Pyne S (2010) Sphingosine 1‐phosphate and cancer. Nat Rev Cancer 10, 489–503. 20555359 10.1038/nrc2875

[3] Johnson KR , Johnson KY , Crellin HG , Ogretmen B , Boylan AM , Harley RA and Obeid LM (2005) Immunohistochemical distribution of sphingosine kinase 1 in normal and tumor lung tissue. J Histochem Cytochem 53, 1159–1166. 15923363 10.1369/jhc.4A6606.2005

[4] Kawamori T , Kaneshiro T , Okumura M , Maalouf S , Uflacker A , Bielawski J , Hannun YA and Obeid LM (2009) Role for sphingosine kinase 1 in colon carcinogenesis. FASEB J 23, 405–414. 18824518 10.1096/fj.08-117572 PMC2630788

[5] Akao Y , Banno Y , Nakagawa Y , Hasegawa N , Kim TJ , Murate T , Igarashi Y and Nozawa Y (2006) High expression of sphingosine kinase 1 and S1P receptors in chemotherapy‐resistant prostate cancer PC3 cells and their camptothecin‐induced up‐regulation. Biochem Biophys Res Comm 342, 1284–1290. 16516161 10.1016/j.bbrc.2006.02.070

[6] Bayerl MG , Bruggeman RD , Conroy EJ , Hengst JA , King TS , Jimenez M , Claxton DF and Yun JK (2008) Sphingosine kinase 1 protein and mRNA are overexpressed in non‐Hodgkin lymphomas and are attractive targets for novel pharmacological interventions. Leuk Lymphoma 49, 948–954. 18452097 10.1080/10428190801911654

[7] Long JS , Edwards J , Watson C , Tovey S , Mair KM , Schiff R , Natarajan V , Pyne NJ and Pyne S (2010) Sphingosine kinase 1 induces tolerance to human epidermal growth factor receptor 2 and prevents formation of a migratory phenotype in response to sphingosine 1‐phosphate in estrogen receptor‐positive breast cancer cells. Mol Cell Biol 30, 3827–3841. 20516217 10.1128/MCB.01133-09 PMC2916408

[8] Pchejetski D , Doumerc N , Golzio M , Naymark M , Teissie J , Kohama T , Waxman J , Malavaud B and Cuvillier O (2008) Chemosensitizing effects of sphingosine kinase‐1 inhibition in prostate cancer cell and animal models. Mol Cancer Ther 7, 1836–1845. 18644996 10.1158/1535-7163.MCT-07-2322

[9] Watson C , Long JS , Orange C , Tannahill CL , Mallon E , McGlynn LM , Pyne S , Pyne NJ and Edwards J (2010) High expression of sphingosine 1‐phosphate receptors, S1P1 and S1P3, sphingosine kinase 1, and extracellular signal‐regulated kinase‐1/2 is associated with development of tamoxifen resistance in estrogen receptor‐positive breast cancer patients. Am J Pathol 177, 2205–2215. 20889557 10.2353/ajpath.2010.100220 PMC2966780

[10] Taha TA , Osta W , Kozhaya L , Bielawski J , Johnson KR , Gillanders WE , Dbaibo GS , Hannun YA and Obeid LM (2004) Down‐regulation of sphingosine kinase‐1 by DNA damage: dependence on proteases and p53. J Biol Chem 279, 20546–20554. 14988393 10.1074/jbc.M401259200

[11] Heffernan‐Stroud LA , Helke KL , Jenkins RW , De Costa AM , Hannun YA and Obeid LM (2012) Defining a role for sphingosine kinase 1 in p53‐dependent tumors. Oncogene 31, 1166–1175. 21765468 10.1038/onc.2011.302 PMC3278571

[12] Pereira NA and Song Z (2008) Some commonly used caspase substrates and inhibitors lack the specificity required to monitor individual caspase activity. Biochem Biophys Res Comm 377, 873–877. 18976637 10.1016/j.bbrc.2008.10.101

[13] Haupt S , Berger M , Goldberg Z and Haupt Y (2003) Apoptosis ‐ the p53 network. J Cell Sci 116, 4077–4085. 12972501 10.1242/jcs.00739

[14] Schuler M , Bossy‐Wetzel E , Goldstein JC , Fitzgerald P and Green DR (2000) p53 induces apoptosis by caspase activation through mitochondrial cytochrome *c* release. J Biol Chem 275, 7337–7342. 10702305 10.1074/jbc.275.10.7337

[15] Tinel A and Tschopp J (2004) The PIDDosome, a protein complex implicated in activation of caspase‐2 in response to genotoxic stress. Science 304, 843–846. 15073321 10.1126/science.1095432

[16] Fava LL , Bock FJ , Geley S and Villunger A (2012) Caspase‐2 at a glance. J Cell Sci 125, 5911–5915. 23447670 10.1242/jcs.115105

[17] Bergeron L , Perez GI , Macdonald G , Shi L , Sun Y , Jurisicova A , Varmuza S , Latham KE , Flaws JA , Salter JC *et al* (1998) Defects in regulation of apoptosis in caspase‐2‐deficient mice. Genes Dev 12, 1304–1314. 9573047 10.1101/gad.12.9.1304 PMC316779

[18] Kumar S (2007) Caspase function in programmed cell death. Cell Death Differ 14, 32–43. 17082813 10.1038/sj.cdd.4402060

[19] Sidi S , Sanda T , Kennedy RD , Hagen AT , Jette CA , Hoffmans R , Pascual J , Imamura S , Kishi S , Amatruda JF *et al* (2008) Chk1 suppresses a caspase‐2 apoptotic response to DNA damage that bypasses p53, Bcl‐2, and caspase‐3. Cell 133, 864–877. 18510930 10.1016/j.cell.2008.03.037 PMC2719897

[20] Ando K , Kernan JL , Liu PH , Sanda T , Logette E , Tschopp J , Look AT , Wang J , Bouchier‐Hayes L and Sidi S (2012) PIDD death‐domain phosphorylation by ATM controls prodeath versus prosurvival PIDDosome signaling. Mol Cell 47, 681–693. 22854598 10.1016/j.molcel.2012.06.024 PMC3444620

[21] Thompson R , Shah R , Liu P , Gupta Y , Ando K , Aggarwal A and Sidi S (2015) An inhibitor of PIDDosome formation. Mol Cell 58, 767–779. 25936804 10.1016/j.molcel.2015.03.034 PMC4458193

[22] Lowe SW , Bodis S , McClatchey A , Remington L , Ruley HE , Fisher DE , Housman DE and Jacks T (1994) p53 status and the efficacy of cancer therapy in vivo. Science 266, 807–810. 7973635 10.1126/science.7973635

[23] Levine AJ and Oren M (2009) The first 30 years of p53: growing ever more complex. Nat Rev Cancer 9, 749–758. 19776744 10.1038/nrc2723 PMC2771725

[24] Brosh R and Rotter V (2009) When mutants gain new powers: news from the mutant p53 field. Nat Rev Cancer 9, 701–713. 19693097 10.1038/nrc2693

[25] Carey LA , Perou CM , Livasy CA , Dressler LG , Cowan D , Conway K , Karaca G , Troester MA , Tse CK , Edmiston S *et al* (2006) Race, breast cancer subtypes, and survival in the Carolina Breast Cancer Study. JAMA 295, 2492–2502. 16757721 10.1001/jama.295.21.2492

[26] Canals D , Jenkins RW , Roddy P , Hernandez‐Corbacho MJ , Obeid LM and Hannun YA (2010) Differential effects of ceramide and sphingosine 1‐phosphate on ERM phosphorylation: probing sphingolipid signaling at the outer plasma membrane. J Biol Chem 285, 32476–32485. 20679347 10.1074/jbc.M110.141028 PMC2952249

[27] Bouchier‐Hayes L and Green DR (2010) Real time with caspase‐2. Cell Cycle 9, 12–13. 20016258 10.4161/cc.9.1.10477

[28] Bouchier‐Hayes L , Oberst A , McStay GP , Connell S , Tait SW , Dillon CP , Flanagan JM , Beere HM and Green DR (2009) Characterization of cytoplasmic caspase‐2 activation by induced proximity. Mol Cell 35, 830–840. 19782032 10.1016/j.molcel.2009.07.023 PMC2755603

[29] Wang S , Konorev EA , Kotamraju S , Joseph J , Kalivendi S and Kalyanaraman B (2004) Doxorubicin induces apoptosis in normal and tumor cells via distinctly different mechanisms. intermediacy of H(2)O(2)‐ and p53‐dependent pathways. J Biol Chem 279, 25535–25543. 15054096 10.1074/jbc.M400944200

[30] Panaretakis T , Laane E , Pokrovskaja K , Bjorklund AC , Moustakas A , Zhivotovsky B , Heyman M , Shoshan MC and Grander D (2005) Doxorubicin requires the sequential activation of caspase‐2, protein kinase Cdelta, and c‐Jun NH2‐terminal kinase to induce apoptosis. Mol Biol Cell 16, 3821–3831. 15917298 10.1091/mbc.E04-10-0862 PMC1182319

[31] Hannun YA and Obeid LM (2011) Many ceramides. J Biol Chem 286, 27855–27862. 21693702 10.1074/jbc.R111.254359 PMC3151029

[32] Chandran S and Machamer CE (2012) Inactivation of ceramide transfer protein during pro‐apoptotic stress by Golgi disassembly and caspase cleavage. Biochem J 442, 391–401. 22129459 10.1042/BJ20111461 PMC3354954

[33] Dent P , Tang Y , Yacoub A , Dai Y , Fisher PB and Grant S (2011) CHK1 inhibitors in combination chemotherapy: thinking beyond the cell cycle. Mol Interventions 11, 133–140. 10.1124/mi.11.2.11 PMC3109860 21540473

[34] Thompson R and Eastman A (2013) The cancer therapeutic potential of Chk1 inhibitors: how mechanistic studies impact on clinical trial design. Br J Clin Pharmacol 76, 358–369. 23593991 10.1111/bcp.12139 PMC3769664

[35] Ma Z , Yao G , Zhou B , Fan Y , Gao S and Feng X (2012) The Chk1 inhibitor AZD7762 sensitises p53 mutant breast cancer cells to radiation in vitro and in vivo. Mol Med Rep 6, 897–903. 22825736 10.3892/mmr.2012.999

[36] Ma CX , Cai S , Li S , Ryan CE , Guo Z , Schaiff WT , Lin L , Hoog J , Goiffon RJ , Prat A *et al* (2012) Targeting Chk1 in p53‐deficient triple‐negative breast cancer is therapeutically beneficial in human‐in‐mouse tumor models. J Clin Investig 122, 1541–1552. 22446188 10.1172/JCI58765 PMC3314455

[37] Johnson ES , Lindblom KR , Robeson A , Stevens RD , Ilkayeva OR , Newgard CB , Kornbluth S and Andersen JL (2013) Metabolomic profiling reveals a role for caspase‐2 in lipoapoptosis. J Biol Chem 288, 14463–14475. 23553630 10.1074/jbc.M112.437210 PMC3656301

[38] Wilson CH , Shalini S , Filipovska A , Richman TR , Davies S , Martin SD , McGee SL , Puccini J , Nikolic A , Dorstyn L *et al* (2015) Age‐related proteostasis and metabolic alterations in caspase‐2‐deficient mice. Cell Death Dis 6, e1597. 25611376 10.1038/cddis.2014.567 PMC4669765

[39] Song T , Zhang X , Zhang L , Dong J , Cai W , Gao J and Hong B (2013) miR‐708 promotes the development of bladder carcinoma via direct repression of Caspase‐2. J Cancer Res Clin Oncol 139, 1189–1198. 23568547 10.1007/s00432-013-1392-6 PMC11824749

[40] Meng XD , Zhou ZS , Qiu JH , Shen WH , Wu Q and Xiao J (2014) Increased SPHK1 expression is associated with poor prognosis in bladder cancer. Tumour Biol 35, 2075–2080. 24092575 10.1007/s13277-013-1275-0

[41] Oliver TG , Meylan E , Chang GP , Xue W , Burke JR , Humpton TJ , Hubbard D , Bhutkar A and Jacks T (2011) Caspase‐2‐mediated cleavage of Mdm2 creates a p53‐induced positive feedback loop. Mol Cell 43, 57–71. 21726810 10.1016/j.molcel.2011.06.012 PMC3160283

[42] Liu Q , Guntuku S , Cui XS , Matsuoka S , Cortez D , Tamai K , Luo G , Carattini‐Rivera S , DeMayo F , Bradley A *et al* (2000) Chk1 is an essential kinase that is regulated by Atr and required for the G(2)/M DNA damage checkpoint. Genes Dev 14, 1448–1459. 10859164 PMC316686

[43] Takai H , Tominaga K , Motoyama N , Minamishima YA , Nagahama H , Tsukiyama T , Ikeda K , Nakayama K , Nakanishi M and Nakayama K (2000) Aberrant cell cycle checkpoint function and early embryonic death in Chk1(−/−) mice. Genes Dev 14, 1439–1447. 10859163 PMC316691

