# Supplementary material for: A role for caspase‐2 in sphingosine kinase 1 proteolysis in response to doxorubicin in breast cancer cells – implications for the CHK1‐suppressed pathway
Source: FEBS Open Bio. 2017 Dec 8;8(1):27–40. doi: 10.1002/2211-5463.12344 (PMC5757171; doi:10.1002/2211-5463.12344)

A role for Caspase 2 in Sphingosine Kinase 1 Proteolysis in Response to Doxorubicin in Breast Cancer Cells: Implications for the CHK1-Supressed Pathway*

Brittany L. Carroll[1], Joseph Bonica[1] Achraf A. Shemseddine[1], Yusuf A. Hannun[1], Lina M. Obeid[1,2*]

1 Stony Brook Cancer Center and the Department of Medicine, Stony Brook University, Health Sciences Center, Stony Brook, New York, USA.

2 Northport Veterans Affairs Medical Center, Northport, New York 11768

Supplemental Figure Legends

Figure S1. *Caspase 2 is required for SK1 proteolysis*
(A) MEFS were treated with 0.2uM doxorubicin for 24h and then harvested in RLT buffer, prepared for quantitative reverse transcriptase-PCR, with enzyme expression normalized to ß-actin expression for each reaction in triplicate. (B) MEFS were treated with 0.2uM doxorubicin for 24h and then harvested in RLT buffer, prepared for quantitative reverse transcriptase-PCR, with enzyme expression normalized to ß-actin expression for each reaction in triplicate. (C) MEFS were treated with 10uM cyclohexamide for the indicated times, harvested in RIPA buffer, and total cell lysate analyzed by western blot for the proteins indicated. (D) MEFS were treated with 0 or 20 J/m^2 for 24h and then incubated with C_{17}-sphingosine for 15 minutes. Following incubation, MEFS were harvested for sphingolipidomic analysis by liquid chromatography mass spectrometry (LC/MS) and the C_{17}-containing S1P was normalized to the amount of lipid phosphate for each sample (n=3 *p<0.05 by Two-Way ANOVA).

Figure S2. *CERT cleavage by Caspase 2 is deregulated in mutant p53 TNBC*
(A). MCF7 and MDA-MB-231 cells were treated with 0 or $20J/m^2$ for 24h harvested in RIPA buffer, and total cell lysate was analyzed by western blot for the proteins indicated.

Figure S3. *Loss of SK1 has no effect on cell viability in MCF7 breast cancer cells in combination with doxorubicin*
(A) MCF7 cells were transfected with SK1 siRNA (20nM). Sixty hours after transfection, cells were treated with 0.8uM doxorubicin for 24 hours, and MTT assay was performed as in "Materials and Methods" to assess cell viability.

Figure S4. *Ceramide levels are not significantly affected by loss of CHK1*
(A) MDA-MB-231 cells were transfected with CHK1 siRNA (20nM). Forty-eight hours after transfection, cells were treated with 0.8uM doxorubicin and then harvested for sphingolipidomic analysis by liquid chromatography mass spectrometry (LC/MS) to measure total ceramide (n=3).

Figure S5. ***Inhibition of caspase 2 modestly restores SK1 protein levels upon activation of the CHK1-suppressed cell death pathway***

(A) MDA-MB-231 cells were treated with 0.3 uM AZD7762 and 20 uM Z-VDVAD-FMK or DMSO 2 hours prior to being treated with 0.8 uM doxorubicin or DMSO. After 24 hours, cells were harvested in RIPA buffer, and total cell lysate was analyzed via western blot for the indicated proteins.

(B) Densitometry measures of western blot bands, expressed as a normalized ratio of SK1 to actin band intensity (n=3). *= p<0.05 in comparison to negative control. += p<0.05, ++= p<0.01 in comparison to positive, doxorubicin treated control.

Figure S6. *MCF-7 cells affects cell death rates after 36 hours of treatment.*

(A). MCF-7 cells were treated with 0.8 uM of doxorubicin or vehicle, and a Trypan Blue assay was conducted at the times indicated. Cells were counted via a hemacytometer. Results are percentage of live cells in sample (n=3, *p<0.05 my Student's T-test).

FIGURE S1

S1A

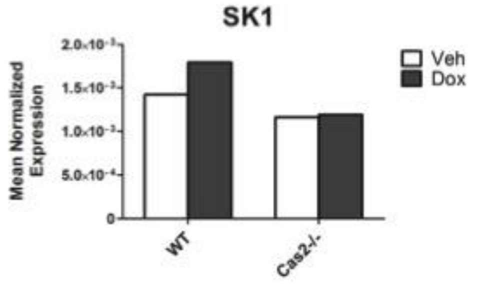

S1B

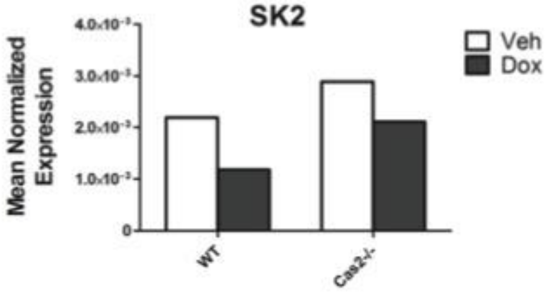

S1C

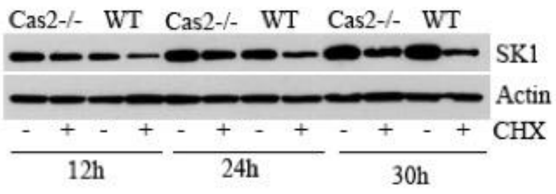

S1D

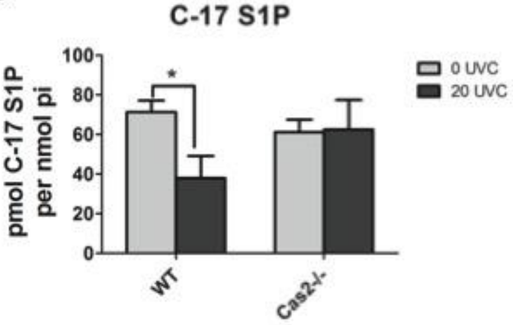

FIGURE S2

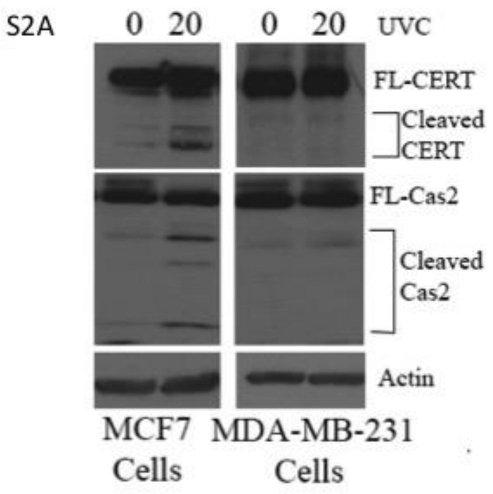
S2A
0
20
0
20
UVC
FL-CERT
Cleaved
CERT
FL-Cas2
Cleaved
Cas2
Actin
MCF7
Cells
MDA-MB-231
Cells

FIGURE S3

S3A

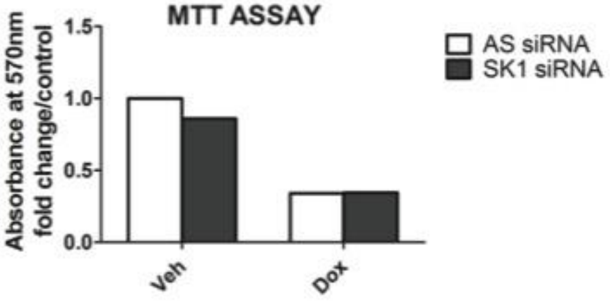

FIGURE S4

S4A

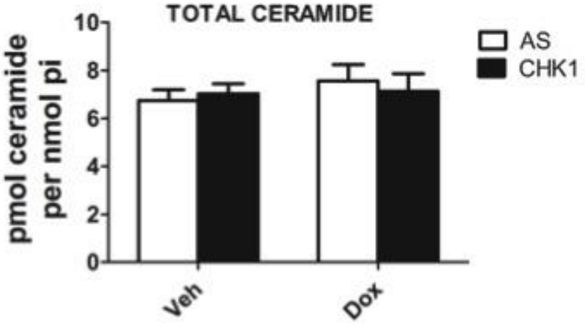

FIGURE S5

S5A

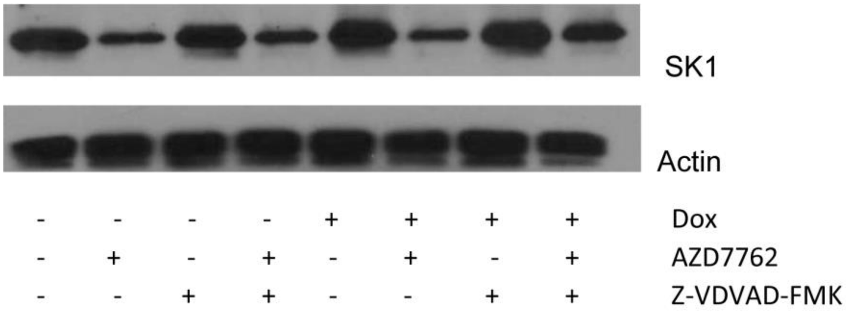

S5B

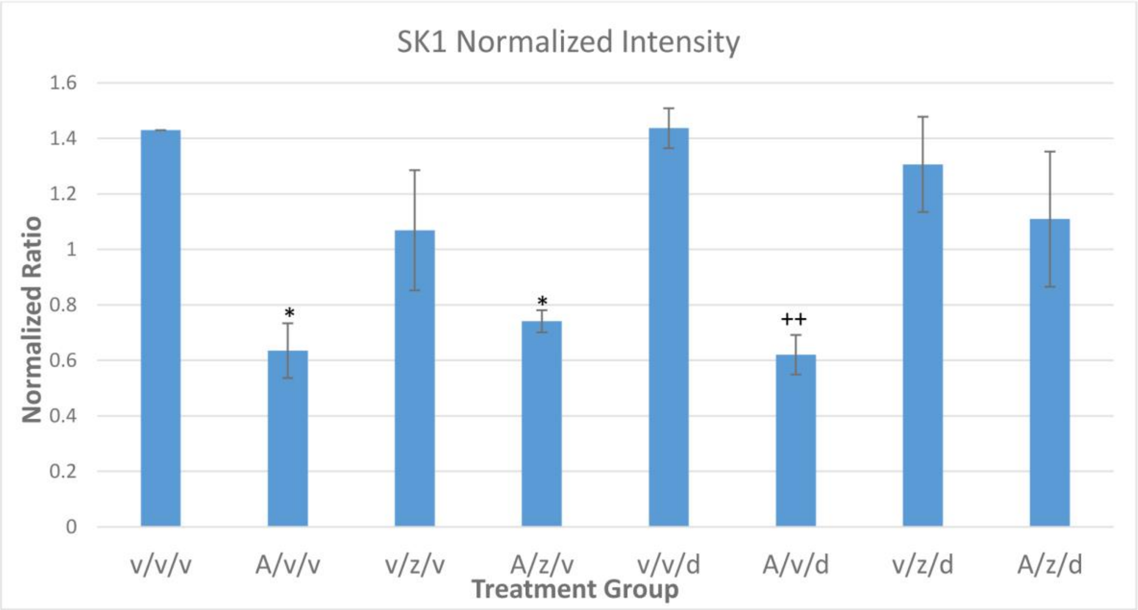

FIGURE S6

S6A

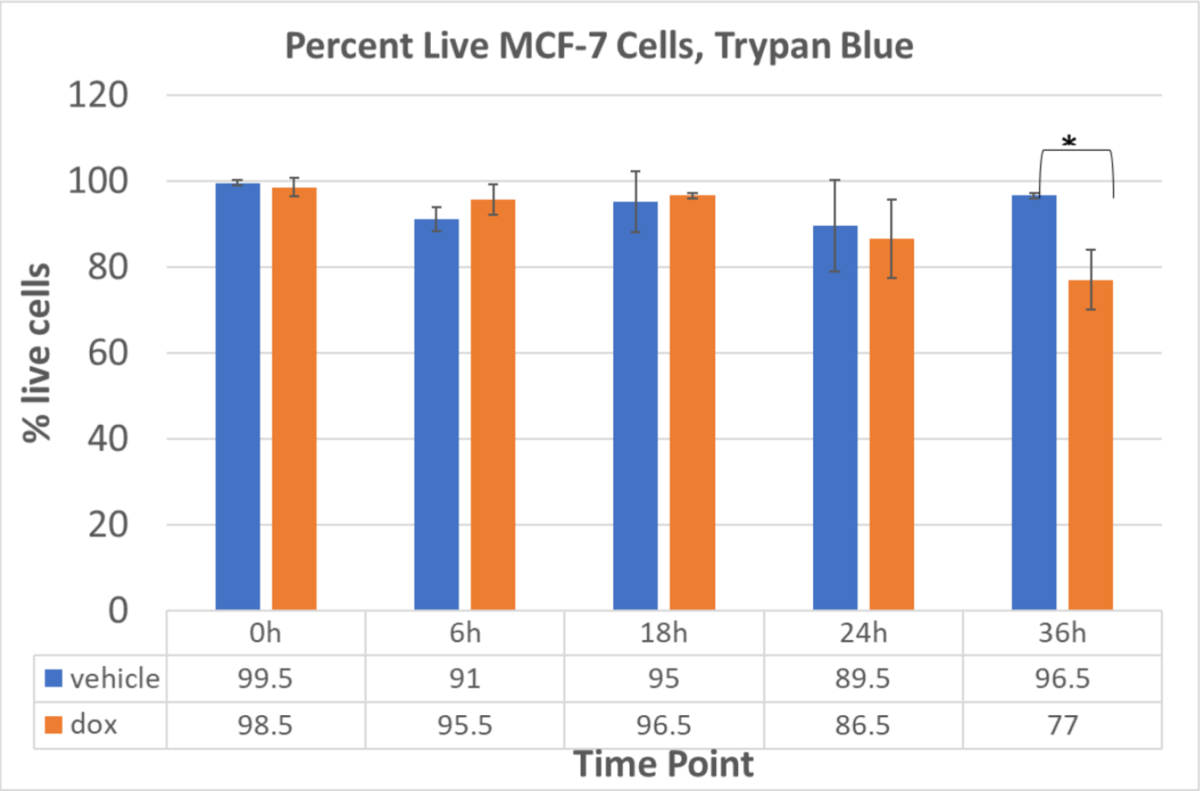

Supplement: Supplementary file 1 — Fig. S1. Caspase 2 is required for SK1 proteolysis. Fig. S2. CERT cleavage by caspase 2 is deregulated in mutant p53 TNBC. Fig. S3. Loss of SK1 has no effect on cell viability in MCF7 breast cancer cells in combination with doxorubicin. Fig. S4. Ceramide levels are not significantly affected by loss of CHK1. Fig. S5. Inhibition of caspase 2 modestly restores SK1 protein levels upon activation of the CHK1‐suppressed cell death pathway. Fig. S6. MCF‐7 cells affects cell death rates after 36 h of treatment. [file FEB4-8-27-s001.pdf]
